# Hypertension in postmenopausal Indonesian Women: fluctuating Body Mass Index as a potential predictor of decreasing blood pressure

**DOI:** 10.4314/ahs.v22i2.30

**Published:** 2022-06

**Authors:** W Riyadina, E Rahajeng, Y Turana, N Kodim

**Affiliations:** 1 National Research and Innovation Agency; 2 School of Medicine and Health Sciences, Atma Jaya Catholic University of Indonesia; 3 Faculty of Public Health University of Indonesia

**Keywords:** Fluctuating, BMI, BP, Postmenopausal, Indonesia

## Abstract

**Background:**

The burden of Hypertension (HT) [P1][YT2] in Indonesian postmenopausal women has increased over the past years. Obesity is the most prevalent risk of HT among postmenopausal women.

**Objective:**

This study evaluates the relationship of fluctuating BMI with BP in postmenopausal women in Bogor, Indonesia.

**Methods:**

This longitudinal study acquired secondary data from a previous study of the “Cohort Study of NCD’ Risk Factors” along with a two-year follow-up. Data were analyzed from a total of 888 postmenopausal women aged ≥ 25 years[P3][wr4]. BMI and BP fluctuations were calculated from baseline BMI at the first visit (T1) until the observation period (T7). The significance of panel analysis at p value < 0.05.[P5][wr6]

**Results:**

The fixed-effect model showed a significant correlation between BMI changes with the changes in SBP and DBP and fluctuating BMI with SBP. After adjustment for physical activity, 1 kg of weight gain will increase SBP and DBP in normotensive, controlled, and uncontrolled hypertensive individuals. After adjusting for smoking, BMI reduction by 1% would lower the SBP as much as 2–3 mmHg compared to a stable BMI.

**Conclusions:**

Fluctuating BMI was a predictor in decreasing BP in postmenopausal women, so it could be used to control HT.

## Introduction

Obesity in postmenopausal women above forty is caused by a decrease in female hormones and an increase in belly fat.^1^ Moreover, it has been reported that 65–75%hypertension is attributable to being overweight.^2^ The 2018 National Health Research report stated that the prevalence of hypertension in the Indonesian population is still relatively high, at 34.1%.^3^ Moreover, the selective prevalence of hypertension in postmenopausal elderly is 58%,%^4^, women of all ages 36.9%^3^, and menopausal women 52,4%.^5^ In 2018, the prevalence of undiagnosed hypertension in Indonesia was 74%.^3^ The May Measurement Month (MMM) survey conducted in 34 provinces in Indonesia found that 30.0% of the population experienced hypertension. 6 Among the 47.4% of hypertensive individuals on antihypertensive medication, 78.0% had uncontroled blood pressure (BP).^6^

Hypertension is, principally, a preventable disease. Health behaviour in persons with hypertension comprise of a dietary approach to stop hypertension or DASH consumption. 7 Health behaviour is the result of interaction between personal and environmental factors.^8^ Postmenopausal repercussions frequently occur in older women 2–5 years after menopause. It is the last biological process of the menstrual cycle that happens due to the decrease of ovarian-produced estrogen hormone levels.^9^ Weight gain often arises in women when they experience this postmenopausal phase.^10^ Obesity in postmenopausal women is attributed to both genetic and environmental factors.^11^ Postmenopausal women are susceptible to diseases attributable to their past lifestyles, such as hypertension and diabetes.^12^ Postmenopausal women have a doubled risk of weight gain and increased blood pressure due to decreasing estrogen hormones and increasing age, and this is sometimes referred to as the degenerative process.

The ability of postmenopausal women to maintain and control blood pressure is the basis of this study. Consequently, advanced research and further study in correlation to fluctuating Body Mass Index (BMI) and blood pressure are needed to contribute to the prevention and maintenance of hypertension in postmenopausal women. Fluctuation in BMI substantially influences fluctuations in blood pressure. It can be utilized as a predictor of increasing blood pressure in normotensive cases and a prognostic determinant in patients with hypertension. Though rarely conducted, BMI and blood pressure fluctuations during observation can be explained using a longitudinal and qualitative design study and tested with data panel analysis. 13 This study determines the correlation of fluctuating BMI and blood pressure as an effort to prevent hypertension and maintain blood pressure in postmenopausal women in Bogor, Indonesia.

## Methods

### Study design

**Fig. 1:**
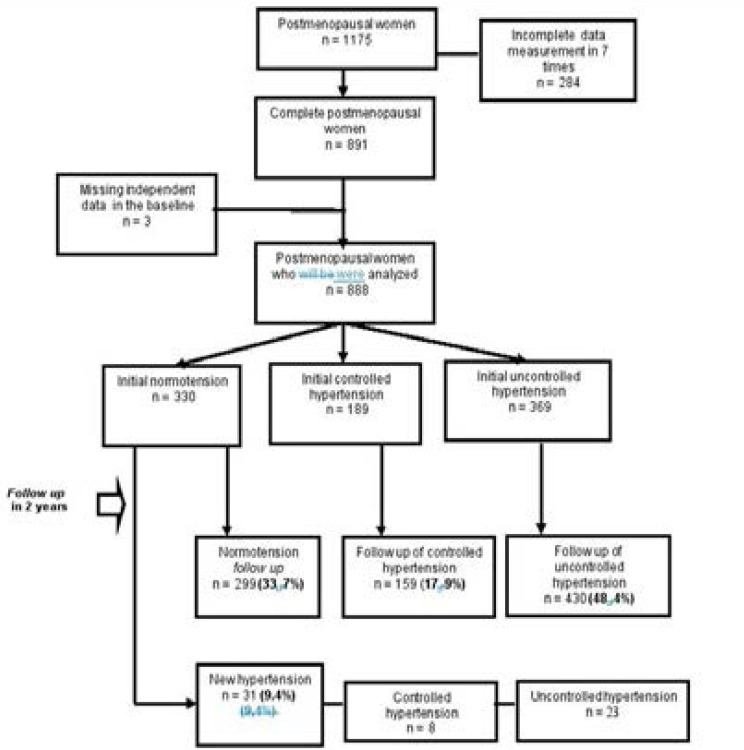
Flow chart of selected data

This study used a mixed-method, which combines two models of longitudinal and qualitative design studies. The study population is postmenopausal women who live in 5 villages: KebonKalapa, BabakanPasar, Babakan, Panaragan, and Ciwaringin. This study analyzed secondary data from “Cohort Study of Non-Communicable Diseases' Risk Factors” in Bogor Tengah district, Bogor City, west of Java. Meanwhile, the sample consists of postmenopausal women whose data collection was complete (no missing data in 7 visits). There were 888 (75.6%) completed data responses from the total study population. The data selection flow is portrayed in the flowchart (Fig. 1).

### Study population and sampling

The data source is secondary data collected from 2011 to 2012 and two-year follow-up data from 2013 to 2014. Data was collected through 2 stages: quantitative (secondary data analysis) and qualitative. Data from postmenopausal women who completed all 7 measurements were selected for this study. The independent variable was fluctuations in BMI, and the dependent variable was the changes in systolic and diastolic pressure.

### Data collection

Blood pressure was determined through measurement of systolic blood pressure (SBP) and diastolic blood pressure (DBP) using calculation of mean blood pressure (average values) in 2 measurements with the nearest difference. The fluctuation in blood pressure was calculated using mean and delta values of 7 repeated measurements. Normotenssion and initial hypertension was determined at 2 measurements, which are T1 (first visit) and T2 (second visit). Follow-up determination of normotension and hypertension (controlled and uncontrolled) was done at the end of observation (T7). The diagnosis of hypertension was made based on two measurements and included blood pressure values (SBP ≥ 140 mmHg or DBP ≥ 90 mmHg) and patient history (information on antihypertensive drug consumption or history of previous hypertension).^14^ Women with a mean SBP of <140 mmHg, mean DBP of <90 mmHg, who never consumed antihypertensive medications, and had no prior history of hypertension were identified as normotensive. Hypertension was defined in women with a previous history of diagnosed hypertension who had a mean SBP of ≥ 140 mmHg and a mean DBP of ≥ 90 mmHg and had no comorbidity of diabetes or coronary heart disease (CHD). women with a history of hypertension along with the comorbidity of diabetes or CHD, were defined as having hypertension if their mean SBP was ≥ 130 mm Hg [P7] and mean DBP ≥ 90 mmHg[P8]. Uncontrolled hypertension was determined in subjects with mean SBP ≥ 140 mmHg and mean DBP ≥ 90 mmHg who had no comorbidities and mean SBP ≥ 130 mmHg and mean DBP ≥ 90 mmHg who had comorbidity of diabetes or CHD.^14^ The study also used standardized methods and calibrated medical equipment. Moreover, the measurement was performed by trained personnel in blood pressure, body weight, and height measurements to calculate BMI precisely. Covariate variables comprised of subject characteristics (age, education, and occupation) behavioral factors (physical activity, smoking habit, and nutrients intake), lipid profiles (HDL, LDL, and triglycerides), blood glucose, and stress.

Intake and nutritional adequacy levels were categorized based on the 2013 Indonesian Balanced nutrition guidelines' standards.^15^ The normal limits of lipid profiles and blood glucose was determined based on the standards recommended by NCEP 2014 and Perkeni 2015.^16^,^17^ Normal lipid profiles and blood glucose was determined using the following limits: total cholesterol <200 mg/dl, LDL <100 mg/dl, HDL>50 mg/dl, triglycerides <150 mg/dl, fasting glucose <126 mg/dl, and postprandial glucose <200 mg/dl. The subjects' physical activity level was categorized based on the Global Physical Activity Questionnaire (GPAQ) instrument.^18^ Smoking habits was determined by the Brinkmen index^19^, and stress was identified using the SRQ instrument.^20^

### Statistical analysis

Changes in BMI were assessed from the difference of weight between measurements (Δ / delta). BMI fluctuations were calculated from mean BMI and delta values of the seven measurements during observation. The initial determination of BMI change was made by conducting measurements of T1 (baseline) and T2 (second visit). Therefore, cut off fluctuating in BMI was determined at 1% based on data distribution in the median limit. BMI fluctuations were classified into three categories; stable, decreasing, and increasing BMI fluctuations. Data analysis used panel tests through fixed-effect modelling by considering the changes in pattern variations of each individual. Quantitative data analysis was performed using Stata for Windows (serial number 401306233465).

## Results

### Participant characteristics

The majority of postmenopausal women in this study were 45–59 years old, unemployed, obese, and experienced hypertension (17.91% suffered from controlled hypertension, and 48.69% from uncontrolled hypertension). The two-year incident rate of hypertension was 5 cases per 100 person-years, and the cumulative incidence was 9.4%.

During the two-year observation period, we found that a total of 299 (33.9%) postmenopausal women in our study were able to maintain their normal blood pressure (normotensive state), whereas 159 (17.9%) women had controlled hypertension, and 430 (48.4%) women had uncontrolled hypertension.

The number of patients with controlled hypertension who were previously determined as having uncontrolled hypertension during this study increased to 34 people. This observation indicates that there was an increase in hypertension control of about 5.77%. The prevalence of controlled hypertension during the two-year observation was 21–33%. Both these findings provide evidence of successful control of hypertension during the two-year observation period. Qualitative results can further explain these outcomes. Knowledge and public attitudes toward hypertension control are quite good, but hypertension control in practice still lacks precision.

### The pattern of change in BMI and Blood Pressure

The pattern of change in BMI is the same between normotensive and hypertensive patients; they increase from the beginning until the end of observation. The BMI changes are about 0.1 – 0.4 units of BMI and is equivalent to 30 – 840 grams. The pattern of change in blood pressure among normotensive individuals and patients with controlled hypertension is the same. In contrast, patients with uncontrolled hypertension experienced decreasing blood pressure. The percentage of controlled hypertension during the two years of observation is 21%–33%. It is still far below the target of the national non-communicable disease prevention and control program, which is 80%.^21^

After controlling for age and physical activity factors, a fitted model of the fixed-effect panel shows that the change in BMI is significantly related to the change of SBP (p=0.001) [Table 1]. The increase of BMI units is the same and is equivalent to 450–460 grams of weight, and increases SBP up to 1.6 mmHg. This result is converted to 1 kilogram of weight gain alongside the increase of SBP, starting from 0.9 mmHg, 2.7 mmHg, and 3.7 mmHg.

**Table 1:** Characteristics of study participants

Characteristic	N-888	Percentage (%)
Age (years)		
40–44	33	3.72
45–59	738	83.11
60–64	117	13.18

Education		
Low	531	59.80
Medium-high	357	40.20

Occupation		
Employment	308	34.68
No employment	580	65.32

Obesity		
No Obese	388	43.69
Obese	500	56.31

Blood pressure status		
Normotension	299	33.67
Controlled hypertension	159	17.91
Uncontrolled hypertension	489	48.42

A fixed-effect model applied to the change in BMI and change in DBP shows a significant correlation after controlling the age, physical activity, and triglyceride levels. For postmenopausal women, an increase in one BMI unit is equivalent to 450–460 grams of body weight alongside increase/span>d DBP from 0.4 mmHg, 0.6 mmHg, and 0.6 mmHg, respectively for cases of normotensive, controlled hypertensive, and uncontrolled hypertensive patients. After conversion, this result shows that an increase of 1 kilogram of weight gain occurs alongside an increase of DBP starting from 0.9 mmHg, 1.3 mmHg, and 1.3 mmHg for normotensive, controlled hypertensive, and uncontrolled hypertensive patients, respectively. (Table 2).

**Table 2:** Relationships between the change of BMI and the changes of SBP

Group	Mean β BMI			Confidence Interval (95%)	
	
	Unadjusted	adjusted	p	Lower	Upper
Normotension	0.6	0.7	0.007	0.2	1.2
Controlled HT*	1.1	1.2	0.001	0.5	1.9
Uncontrolled HT*	1.5	1.7	0.001	1.0	2.3

* HT: Hypertension; fixed effect model, adjusted by age and physical activity

### Fluctuations of BMI and Blood Pressure

Systolic BP is more prone to changes than DBP. The changes in DBP tend to be more stable. It is related to hemodynamics that causes an increase in cardiac output^22^ The fluctuations of BMI increases and decreases by 1%, and is significantly related to SBP fluctuations (Table 4), but t is not equivalent to DBP fluctuations (Table 5).

**Table 4:** Relationship between fluctuating in BMI and the change of SBP

	Mean β BMI			Confidence Interval (95%)	
			
Group	Unadjusted	adjusted	p	Lower	Upper
Normotension	-1.6	-1.9	0.027	-3.5	0.2
Controlled HT*	3.7	3.6	0.011	0.8	6.4
Uncontrolled HT*	-2.9	2.7	0.024	-5.1	-0.4

* HT: Hypertension, fixed effect model adjusted by age and smoking habit

**Table 5:** Relationship between fluctuating in BMI and the change of DBP

Group	Mean β BMI			Confidence Interval (95%)	
			
	Unadjusted	adjusted	p	Lower	Upper
Normotension	- 1.1	-1.2	0.031	-2.2	-0.1
Controlled HT*	0.1	0.2	0.782	-1.4	1.9
Uncontrolled HT*	-0.9	0.9	0.133	-2.1	0.3

* HT: Hypertension, fixed effect model adjusted by age

A 1% decrease in BMI lowers SBP by 1.9 mmHg and 2.7 mmHg in each case for normotensive and uncontrolled hypertensive patients, respectively. In other words, after adjusting for smoking, a BMI reduction by 1% would lower the SBP as much as 2–3 mmHg compared to a stable BMI. In a natural sample without any interventions, a 1% fluctuation in BMI shows a small change that is quite sensitive to blood pressure change. The biological mechanism of weight reduction can lead to the decrease of blood pressure as well due to the increasing of bioavailability of nitrate oxide, angiotensin II reduction, and decreased sympathetic nerve activity.^23^

## Discussion

Our findings show a similar BMI fluctuation pattern among normotensive, controlled hypertensive, and uncontrolled hypertensive patients during the observation period. Weight gain in 4 months of observation and measurements had the lowest weight gain (30 grams) and the highest weight gan (840 grams). The weight gain ratio of BMI is 0.45–0.46 (450–460 gram), which means that the increase or decrease in BMI units is proportional to the increase or decrease in body weight by 450–460 grams. Relatively, the weight did not change during the follow-up stage (weight of participants oscillated only by ± 0.4 kg/m2).^24^ Multilevel research proves that weight, BMI, and body fat percentage are the most potent anthropological determinants of blood pressure alterations in the study population. The rate of premenopausal BMI increase and premenopausal episodic weight loss of more than 5 kg have been independently associated with a later age of natural menopause. 25

Qualitative results indicated that patients with controlled hypertension have herbal consumption habits that are believed to maintain health and normal blood pressure. Some widely consumed vegetables that are used as alternative herbal medications include cucumber and squash.^26^ About 80% of the world's population is still dependent on traditional medications, including the use of plant-derived medicines.^27^ Various natural ingredients are thought to lower blood pressure as well.^28^ Cucumber contains many saponins, flavonoid, and polyphenols compounds that have been empirically proven to lower blood pressure. 29 The effect of potassium, calcium, and magnesium of cucumber can play an essential role in supporting the potassium-sodium pumps in the human body.^30^ Other habits among controlled hypertensive patients include doing routine physical activities such as walking and gymnastics together at least 2–3 times a week. Research results state that a 30-minute walk leads to decreased diastolic blood pressure in hypertension patients at the General Hospital of Kebonjahe.[P9][wr10]^31^ The respondents were less in sports activities or not ideal (<3 times per a week and 30 minutes) at risk of developing hypertension by 4.73 times compared to people who are active in sports..31 When performed frequently, exercise improves heart function, increases muscle strength, burns fat, and can have various other benefits on the body.^32^

Escalated change in BMI units increases SBP more significantly than DBP. This finding occurred in all three patient groups (normotensive, controlled hypertensive, and uncontrolled hypertensive patients). DBP was proven to be more stable than SBP. Changes in blood pressure rise indicate a smaller β coefficient in normotensive states than hypertensive states (both controlled and uncontrolled) as seen as 0.7 vs 1.2; 1.7 for SBP and 0.4 vs 0.6; 0.6 for DBP. This result can be converted to show [P11]an increase of BMI units equal to 1 kg of weight and occurs alongside an increase in SBP by 1.5 mmHg, 2.7 mmHg, and 3.7 mmHg for normotensive, controlled hypertensive, and uncontrolled hypertensive patients, respectively.

In comparison, the same increase of BMI occurs along with an increase of DBP by 0.9 mmHg for normotensive individuals and 1.3 mmHg for both the controlled and uncontrolled hypertension patient groups. The results show that SBP is more sensitive to change than DBP. The study results state that over five years, an increase of 1.8 BMI units in women was estimated to increase SBP by 2.3 mmHg and 1.8 mmHg. Previous literature has suggested that women experience an annual average increase in blood pressure; a cross-sectional found a 1.5 mmHg increase, and a longitudinal study found a 1.3 mmHg increase. 33

Changes in cardiac output (CO), peripheral vascular resistance, blood pressure,, and systemic vascular resistance (SVR) are factors linked to obesity and blood pressure. Increased BMI is associated with higher body fluid volume, peripheral resistance (such as hyperinsulinemia, cell membrane changes, and renin-angiotensin changes-factors that influences functional narrowing and structural hypertrophy), and cardiac output.^34^ Increased BMI is also related to increased visceral fat, leading to increased leptin and insulin resistance, poor lipid profiles, and increased development of atherosclerosis and chronic renal failure.^35^

The results prove that fluctuating BMI is significantly related to change in SBP, but not in DBP. Research on African-American women suggests that weight loss affects the fluctuation of blood pressure. The study reported randomization to 60 months; mean SBP increased to a similar degree for the weight gain group (4.2 ± SE=0.6 mm Hg; p<0.001) and stable weight group (4.6 ± 1.1 mm Hg; p<0.001), but SBP did not rise in the weight loss grop (1.0 ± 1.7 mm Hg, p=0.53). DBP was unchanged for all groups at 60 months.^36^ In postmenopausal women with a normotensive state, fluctuating BMI may lower SBP by 1.9 mmHg and DBP by 1.2 mmHg. In uncontrolled hypertension patients, the decrease in BMI decreased SBP by 2.7 mmHg. Qualitative data supports these results. Factors influencing BMI fluctuations such as physical activity, religious activity/worship, and stable psychological and social conditions are more likely to be performed by and occur in normotensive rather than hypertensive individuals. In this study, the 1% delta change criteria in BMI categories were stable, and its fluctuations were attributable to the short follow-up time (4-month interval). The distribution of data showed small changes, and the sample was in a natural condition without any intervention. Thus, a 1% change was adequately sensitive due to a significant relationship between fluctuating BMI and the fluctuation of SBP. Another study found that a 5% decrease in BMI was significantly associated with reduced BP- this result was based on a sample with different characteristics of overweight and obese groups, groups of elderly over 65 years, a longer follow-up time (in years), a different study design, and utilized interventions to attain a more significant impact).^23^

The mechanism of decreasing arterial vascular stiffness related to weight loss remains unclear. Increased nitrate oxide bioavailability, angiotensin II reduction, decreased sympathetic neurological activity, and other factors may contribute to the change in arterial stiffness observed and weight loss.^23^ results from this study support the relationship independently. The changes in BMI cause changes in SBP and DBP among both women and men. The increase in BMI leads to the risk of hypertension.^37^ Hypertensive women had significantly higher BMI value than non-hypertensive women.^38^ These observations raise the possibility that the impact of obesity-related neural activity on arterial pressure is modified by environmental and genetic factors, including race and gender.^39^. Observational studies among normal, overweight, and obese men and women also stated that a 5% weight gain per kilogram increases hypertension risk. A weight gain by 4–6 kilograms increases the risk of hypertension by 1.25 times and 1.65 times for 7 kilograms of weight gain and above compared to the control group. The onset of hypertension in peri-[P12]and early postmenopausal women was strongly related to an increase in body weight despite controlling for initial body weight, reported physical activity, and hormonal use[YT13].^40^

This study's strength is its longitudinal design using 7-point data (visit) and panel analysis methods so that inter-and intra-individual variations were taken into account. The relationship between the prognosis of the diseases can be more greatly appreciated due to the longitudinal design study. This study limitations are the use of secondary data and the implementation of a 4-month measurement interval, which restricts the observation of weight changes in adult women. The baseline postmenopausal determination was not known for the study sample of menopausal status before the study began.

## Conclusion

This study has found a relationship between BMI change and the change of blood pressure in postmenopausal women. The fluctuations in BMI can be used to predict decreasing BP and hence can be utilized in risk factor surveillance for non-communicable diseases. Fluctuating BMI is a potential predictor of decreasing blood pressure and could be used to control hypertension.

## Figures and Tables

**Table 3:** Relationships between the change of BMI and the change of DBP

	Mean β BMI			Confidence Interval (95%)	
			
Group	Unadjusted	adjusted	p	Lower	Upper
Normotension	0.6	0.4	0.007	0.1	0.7
Controlled HT*	0.6	0.6	0.007	0.2	1.0
Uncontrolled HT*	0.5	0.6	0.001	0.3	0.9

* HT: Hypertension; fixed effect model adjusted by age and trygliceride
